# Magnetic Nanoparticles to Unique DNA Tracers: Effect of Functionalization on Physico-chemical Properties

**DOI:** 10.1186/s11671-021-03483-5

**Published:** 2021-02-06

**Authors:** Anuvansh Sharma, Jan Willem Foppen, Abhishek Banerjee, Slimani Sawssen, Nirmalya Bachhar, Davide Peddis, Sulalit Bandyopadhyay

**Affiliations:** 1 Department of Materials Science and Engineering, Norwegian University of Science and Technology (NTNU), 7491 Trondheim, Norway; 2 Department of Water Science and Engineering, IHE Delft Institute for Water Education, PO Box 3015, Delft, The Netherlands; 3 Dipartimento di Chimica e Chimica Industriale, Università di Genova, Genoa, Italy; 4 Istituto di Struttura della Materia – CNR, Area della Ricerca di Roma1, 00015 Monterotondo Scalo, RM Italy; 5 Department of Chemical Engineering, Indian Institute of Technology Jodhpur, Jodhpur, 342037 India; 6 Department of Water Management, Delft University of Technology, PO Box 5048, 2600 GA Delft, The Netherlands; 7 Department of Chemical Engineering, Norwegian University of Science and Technology (NTNU), 7491 Trondheim, Norway

**Keywords:** Hydrological tracers, Magnetic iron oxide nanoparticles, DNA encapsulation, Phase transfer, Silica nanoparticles

## Abstract

**Abstract:**

To monitor and manage hydrological systems such as brooks, streams, rivers, the use of tracers is a well-established process. Limited number of potential tracers such as salts, isotopes and dyes, make study of hydrological processes a challenge. Traditional tracers find limited use due to lack of multiplexed, multipoint tracing and background noise, among others. In this regard, DNA based tracers possess remarkable advantages including, environmentally friendly, stability, and high sensitivity in addition to showing great potential in the synthesis of ideally unlimited number of unique tracers capable of multipoint tracing. To prevent unintentional losses in the environment during application and easy recovery for analysis, we hereby report DNA encapsulation in silica containing magnetic cores (iron oxide) of two different shapes—spheres and cubes. The iron oxide nanoparticles having size range 10–20 nm, have been synthesized using co-precipitation of iron salts or thermal decomposition of iron oleate precursor in the presence of oleic acid or sodium oleate. Physico-chemical properties such as size, zeta potential, magnetism etc. of the iron oxide nanoparticles have been optimized using different ligands for effective binding of dsDNA, followed by silanization. We report for the first time the effect of surface coating on the magnetic properties of the iron oxide nanoparticles at each stage of functionalization, culminating in silica shells. Efficiency of encapsulation of three different dsDNA molecules has been studied using quantitative polymerase chain reaction (qPCR). Our results show that our DNA based magnetic tracers are excellent candidates for hydrological monitoring with easy recoverability and high signal amplification.

**Graphic Abstract:**

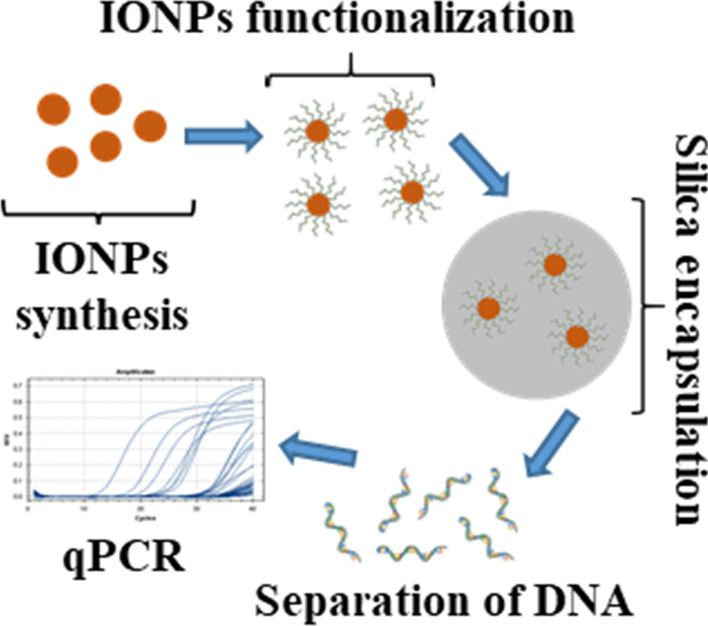

## Introduction

Tracer tests are a widely used technique to investigate flow paths and travel times of solutes, particulates and contaminants in environmental waters [1, 2]. However, there are limited number of potential tracers available to hydrologists to study these dynamic processes. Traditional tracers, such as natural dissolved salts, stable isotopes, bromide, and fluorescent dyes [1, 3], have been reported to suffer from interference of background noise, high analysis costs, potential environmental contamination, signal contamination among others. Furthermore, application of tracers at larger scales/solvent volumes results in dilution effects that pose issues with their detection limits thereby becoming an important constraint.

DNA tracers have been reported to be specific, (i.e. bearing unique identifiers that do not suffer from interference due to background noise) environmentally friendly, ultrasensitive to detection and multiplex capable (i.e. enabling concurrent usage of a large number of species at a given time) [4–7]. Importantly, DNA tracers can be repetitively and thus exponentially amplified, using quantitative polymerase chain reaction (qPCR), theoretically pushing the detection sensitivity down to one molecule [8, 9]. Hence, tracers containing DNA require a detection amount that is orders of magnitude lower than that of traditional tracers.

Foppen et al. and Sharma et al. were the first to test DNA as an applied tracer in surface water. Foppen et al. [10–12] compared the breakthrough and recovery rates of six different free DNA tracers and a conservative tracer in several stream channel experiments. These DNA tracers showed consistent advective–dispersive transport properties, but low mass recovery for the free DNA tracers (3–53%) compared with the conservative tracer (67–106%) [10]. This mass loss of the free DNA tracers was attributed to adsorption onto sediment particles, decay and/or biological uptake by microorganisms. Encapsulating DNA inside a protective shell was therefore suggested in order to enhance the longevity of the tracer.

To our knowledge, there are four types of encapsulated DNA tracers reported in the literature—(1) DNA wrapped and stabilized by a combination of polyamidoamine (PAA), a cationic homopolymer and a poly(ethylene) glycol (PEG)-poly(amidiamine) (PAA)-PEG copolymer (Garnett particle) [13], (2) iron oxide nanoparticles (IONPs) plus DNA, encapsulated by polylactic acid (PLA) (Sharma particle) [12], (3) silica core, surrounded by a layer of DNA and protected again by silica (Paunescu particle) [14, 15] and (4) magnetite with DNA wrapped around them and protected by silica cover (Puddu particle) [4]. In case of the latter two, DNA is encapsulated within silica shells, that have been shown to be heat and radical resistant, whereby isolating the DNA completely from the environment. However, in these studies, a single type of DNA has been used for fabrication of the tracer. More importantly, there is no report of how the magnetic properties of the tracers change during the functionalization process. This is important to understand since the rationale behind incorporating magnetic nanoparticles (MNPs) lies in fast and easy magnetic separation, better sample handling, absence of sample volume limitations and up-concentration. Studies so far have focussed only on magnetite as the core of the tracer. Particle size and shape of the IONPs among other factors have a crucial role in determining the magnetic properties of the tracers and in turn their magnetic harvesting for separation and analysis.

To obtain a precise control over the particle’s size, shape and morphology and eventually, their magnetic properties, IONPs can be synthesized using thermal decomposition route. This refers to decomposition of iron precursors such as acetylacetonate, stearate, oleate or pentacarbonyl in the presence of stabilizing ligands in an organic solvent at high temperatures [16–19]. The finer control over particle size originates from temporal separation of nucleation and growth windows that promotes narrower size distributions in comparison to the more prevalent co-precipitation route. However, the obtained nanoparticles (NPs) in the former case are in organic phase that limits their use in environmental and biomedical applications [20–22]. Several reports have shown that the phase transfer of these NPs may be achieved using specific ligands such as tetra methyl ammonium hydroxide (TMAH), hexadecyltrimethylammonium bromide (CTAB), poly-ethylene glycol (PEG), sodium citrate (Na-citrate) and so on, guided by the synthesis route and application downstream [23–25]. Phase transfer however influences subsequent functionalization steps of tracer synthesis, such as binding of the DNA to the core, tracer encapsulation, etc. In cases, where the effect of particle’s size, shape and morphology on functionalization needs to be investigated, selecting the same phase transfer methodology for all the particle types deems necessary. In order to achieve this, developing repeatable and tunable protocols becomes utmost important.

Further, functionalization steps can impact the magnetic properties of the final tracer particle, whereby affecting their applicability downstream. In fact, the molecular coating can strongly influence surface magnetism of the particles, altering the magnetic properties [26, 27]. An understanding of how the physico-chemical properties vary subsequently with the functionalization route is believed to be a precursor in understanding the final functional properties of the tracers. However, such reports are missing in literature. Herein, we report a robust approach to synthesize spinel IONPs via co-precipitation and thermal decomposition routes, followed by functionalization with three different types of double-stranded DNA (dsDNA). The type of the dsDNA used for functionalization provides for the uniqueness of the tracer. Thereafter, we silanize the DNA functionalized IONPs to encapsulate the DNA inside a protective shell, thereby producing customizable, hydrological tracers bearing unique DNA tracer moieties. Our work reports, for the first time, how the magnetic properties alter along the functionalization route—from IONPs to DNA based magnetic tracers, with an aim to understand structure-properties relations for the tracers.

## Method Section

Iron(II)chloride tetrahydrate (FeCl_2_.4H_2_O, ≥ 99%), 1-octadecene (ODE, technical grade 90%), oleic acid (OA, technical grade 90%), sodium citrate dihydrate (Na-citrate, ≥ 99%), tetramethylammonium hydroxide solution (TMAH, ACS reagent) and tetraethyl orthosilicate (TEOS, reagent grade 98%) were purchased from Sigma-Aldrich® (Schnelldorf, Germany). Cetyltrimethylammonium bromide (CTAB, ≥ 99%) and iron(III)chloride hexahydrate (FeCl_3_.6H_2_O, ≥ 99%) were bought from Acros Organics® (Geel, Belgium). Trimethoxysilylpropyl-N,N,N-trimethylammonium chloride (TMAPS, in 50% methanol) and sodium oleate (> 97%) were purchased from abcr® (Karlsruhe, Germany) and Tokyo Chemical Industry (TCI, Japan), respectively. Ammonium hydroxide solution (25 wt%) was bought from Merck Life Sciences AS. The 3 dsDNA namely, T21, GM5 and GM6 were supplied by Biolegio B.V. (Nijmegen, Netherlands) and further details regarding the dsDNA can be found in the Additional information 1 (SI, Materials and Methods). All the chemicals were used as received without any further purification or modification. All solutions were prepared using distilled de-ionized water (MilliQ water), having a resistivity ~ 18.2 MΩ/cm at 25 ℃, taken from Simplicity® Millipore (Darmstadt, Germany) water purification system.

### Synthesis of IONPs

#### Co-precipitation

The synthesis process has been adapted from previously reported studies and involves weighing 84.6 mg of MilliQ water in a beaker and adding 15.4 mL of 25% (wt) NH_4_OH solution to prepare a 1 M solution [4]. Separately, 8.0 g of FeCl2.4H2O and 21.6 g of FeCl_3_·6H_2_O were weighed and dissolved in 100 mL MilliQ water in a volumetric flask. 10 mL of this solution was added to 100 mL of 1 M ammonia solution dropwise (using a burette), the reaction being kept under vigorous stirring. The NPs were obtained using magnetic separation by several washing cycles with water. These particles have been referred to as ‘Mag’ in this report.

#### Thermal Decomposition

Spherical and cubic IONPs were synthesised using thermal decomposition of Fe oleate precursor at elevated temperatures in inert (argon) atmosphere. The temperature of the reaction was raised using a temperature controller (MRC® Heating mantle 100 mL with analog control and Digital 4 programs × 16 segment programmer) for precise control of the heating rate.

##### Iron Oleate

The iron-oleate complex was prepared using our previously reported protocol [28]. In essence, 5.40 g of FeCl_3_·6H_2_O and 18.25 g of sodium oleate were dissolved in a mixture of solvents consisting of 40 mL ethanol, 30 mL deionized water and 70 mL hexane, in 250 mL round bottom flask. The resulting solution was refluxed at 70 °C for 4 h under vigorous stirring using a bar magnet. The resulting dark red organic phase, containing iron oleate complex, was transferred to a separator funnel and washed three times with MilliQ water. The remaining hexane solution was evaporated in a Heidolph rotary evaporator (*T* = 70 °C) to yield a highly viscous dark red liquid of iron oleate.

##### Iron Oleate: Spheres (SNPs)

The synthesis protocol, adapted and modified from previous works conducted by our group, involved mixing 1.6 g of iron oleate, 600 µL OA and 25 mL 1-octadecene (ODE) in a three-necked glass reactor, placed over a heating mantle fitted with a cooling water condenser [28]. The reaction was carried out under argon atmosphere. The temperature of the reactor was ramped from room temperature to 320 ℃ at 3 ℃/min. The reaction was maintained at 320 ℃ for 45 min after which the solution was cooled down to room temperature. NPs were then washed using hexane and precipitated out using a mixture of isopropanol and hexane. The particles were magnetically separated and washed thrice using acetone before finally redispersing in a known volume of toluene.

##### Iron Oleate: Cubes (CNPs)

Adapted and modified from our group’s work, in a typical synthesis, 0.833 g of iron oleate, 213 mg sodium oleate and 14 mL ODE were heated in a three-necked glass reactor placed on a heating mantle, fitted with a cooling water condenser, to 325 ℃ at 2.8 ℃/min under argon atmosphere [29]. The reaction was maintained at 325 ℃ for 45 min after which the NPs were cooled to room temperature. NPs were then washed using hexane and precipitated out using a mixture of isopropanol and hexane. The particles were magnetically separated and washed thrice using acetone before finally redispersing in a known volume of toluene.

### Growth Kinetics

We characterized the growth of SNPs obtained from the decomposition of iron oleate precursor in ODE. Small sample volumes were taken at defined time intervals and analysed using transmission electron microscopy (TEM) and Fourier transform infrared (FTIR) spectroscopy without further cleaning.

We have developed a mechanistic model and carried out kinetic Monte Carlo simulation to understand the growth kinetics of the NPs following our previous work [30]. Our simulation data matched the mean of the particle size distribution (PSD) obtained from analysing the TEM images of SNPs presented in this work. The details of the simulation are reported in the Additional information 1 (Sect. 2).

### Phase Transfer and Functionalization of IONPs

Phase transfer of SNPs and CNPs was carried out using either CTAB, Na-Citrate or TMAH. The detailed procedure can be found in the Additional information 1 (Sect. 3). Once the NPs were successfully phase transferred, they were redispersed either in MilliQ water or isopropanol before further analysis or functionalization. For Mag, direct functionalization was carried out, since the NPs were already dispersed in aqueous phase.

A known amount of IONPs in water (Mag, SNPs and CNPs) were taken and cleaned thrice with isopropanol before finally dispersing in 1 mL isopropanol in a 1.5 mL Eppendorf tube. 10 µL of TMAPS was added to the IONP solution and put on overnight shaking. The samples were collected afterwards, cleaned thrice with isopropanol and redispersed in 1 ml isopropanol.

In a process to functionalize IONPs with dsDNA, a known quantity of IONPs functionalized with TMAPS (Table 1) was added to a predetermined amount of dsDNA (optimized through preliminary experiments) in a 1.5 mL Eppendorf tube. The solution was kept at rest and allowed to react for 4 min. The DNA functionalised NPs were then magnetically separated and cleaned three times with MilliQ water and finally redispersed in 0.5 mL MilliQ water.

**Table 1:** Showing the amount of IONPs taken for surface functionalization with TMAPS and dsDNA respectively

	IONP amount (mg)	TMAPS (µl)	IONP amount (mg)	dsDNA (T21, GM5, GM6) (µl)
Mag	50	10	0.75	20
SNPs	20	40	0.36	20
CNPs	10	20	0.30	20

After successful binding of dsDNA, 1 µL TMAPS was added to 0.5 mL NP solution followed by 1 µL TEOS and the samples were left on shaking for 4 h. 8 µL of TEOS was further added to the solution after 4 h and the shaking was continued for 4 days after which the particles were magnetically separated and cleaned thrice with MilliQ and redispersed in known volume of MilliQ water.

### Characterization Techniques

#### Transmission Electron Microscopy (TEM)

High resolution images were taken using the JEOL 2100 transmission electron microscope (Tokyo, Japan) operating at 200 kV. TEM grids were prepared by placing several drops of the dilute solution on a Formvar carbon-coated copper grid (Electron Microscopy Sciences) and wiping immediately with Kimberly-Clark wipes to prevent further aggregation owing to evaporation at room temperature.

#### Attenuated Total Reflection-Fourier Transform Infrared (ATR-FTIR)

The attenuated total reflection-Fourier transform infrared (ATR-FTIR) spectra were obtained using a Vertex 80v vacuum FTIR spectrometer. OPUS software was used to record spectra between 600 and 4000 cm^−1^ using 50 scans at a resolution of 4 cm^−1^. The spectral absorbances were normalized with respect to the highest absorbance value obtained. In case of the growth kinetic studies of SNPs, sample aliquots were taken at defined temperatures during the reaction and the samples were analysed without further cleaning. On the other hand, for the phase transferred samples, NPs were cleaned thrice using MilliQ water before analysis.

#### Dynamic Light Scattering (DLS)

The size distribution and zeta potential of the NPs were measured using a Malvern Zetasizer Nano-ZS instrument (Malvern Instruments Ltd., Worcestershire, UK) and the manufacturer’s own software. The solvent used for all the NPs was MilliQ water.

#### Super Conducting Quantum Interference Device (SQUID) Magnetometry

DC magnetization measurements were performed with superconducting quantum interference device (SQUID) magnetometer (*H*_max_ = 5.5 T, *T* = 5–400 K). The sample, in the form of powder or liquid, was placed in a capsule, in order to prevent any movement of the NPs during the measurements. All the magnetic measurements were normalized by the mass of the magnetic component of the sample. Magnetization measurements were carried out as a function of temperature from 5 to 300 K using ZFC–FC (Zero Field Cooled–Field Cooled) protocols with an applied field of 25 Oe. Room temperature magnetic properties were investigated using a vibrating sample magnetometer (model 10—MicroSense, *H*_max_ = 20 kOe).

#### Thermogravimetric Analysis

The mass of ligand on the IONPs was calculated using thermogravimetric analysis of the samples using the SDT Q600 Thermogravimetric Analyser (TA® Instruments, Delaware, USA) and the manufacture’s inbuilt software.

#### qPCR Analysis

In order to determine DNA, samples in 400 µL MilliQ water were sent to IHE Delft and stored cool upon arrival. Prior to qPCR, samples were dispersed, and vortexed 3 times @ 3000 rpm for 30 s and sonicated for 10 s (Hielscher UP200 St Vial Tweeter sonicator). Then, MilliQ water was added to a total volume of 1000 μL. In order to remove free DNA, not covered by silica, 1.25 μL commercial bleach was added, and the mixture was vortexed for 15 s. After 1–2 min, the particles were magnetically separated, washed twice with MilliQ water, and finally resuspended in 1 mL MilliQ water. This was the stock solution or D0. From D0, four tenfold dilutions (D1–D4) were prepared and DNA concentrations were determined from the two most dilute samples (D3 and D4). Thereto, 20 µL D3 and D4 were taken out and, in order to dissolve particles and release DNA, 1 µL of fluoride buffer (2.3 g of (NH_4_)HF_2_ and 1.9 gm of NH_4_F in 10 mL water), called Buffered Oxide Etch (BOE) was added and allowed to stand for 10 min. From this, 4 μL of D3 or D4 was taken to which 16 μL of PCR mix was added. In case of DNA tracer T21, the PCR mix composed of 2 µL 10X PCR buffer, 0.8 µL MgCl_2_ buffer, 1 µL dNTP mix, 1 µL Taq polymerase, 10.65 µL DEPC treated water, 0.125 µL F-primer and R-primer, and 0.3 µL of probe, composed of a FAM fluorophore and a black hole quencher. In case of the other two tracers, again 4 μL of D3 or D4 was taken and 16 µL of PCR mix was added, composed of 15.75 μL KAPA SYBR FAST qPCR Master Mix (2X) Universal (Kapa Biosystem), 0.125 µL of both F-primer and R-primer. Samples were inserted in a qPCR apparatus (BioRad Mini-Opticon). The protocol of T21 was 3 min at 95 ℃, followed by 40 cycles of a two-step thermal profile (15 s at 95 ℃, 60 s at 60 ℃). The protocol of the other two tracers was 6 min and 40 s at 95 ℃, followed by 41 cycles of a three-step thermal profile (14 s at 95 ℃, 27 s at 58 ℃, 25 s at 72 ℃).

## Results and Discussion

In order to develop DNA based magnetic tracers for hydrology, three important aspects need to be considered—controlling the size and shape of the magnetic core, protection of the DNA (unique tracer moiety) through robust functionalization procedures and understanding how sequential functionalization influences the magnetic properties of the final tracer particles.

### Controlling the Particle Size and Shape

The magnetic cores in the DNA based tracers have been synthesized using co-precipitation and thermal decomposition routes. The IONPs synthesized using co-precipitation are referred to as ‘Mag’, while the spherical and cubic IONPs synthesised using thermal decomposition of Fe oleate precursor are named as ‘SNPs’ and ‘CNPs’ respectively.

**Fig. 1:**
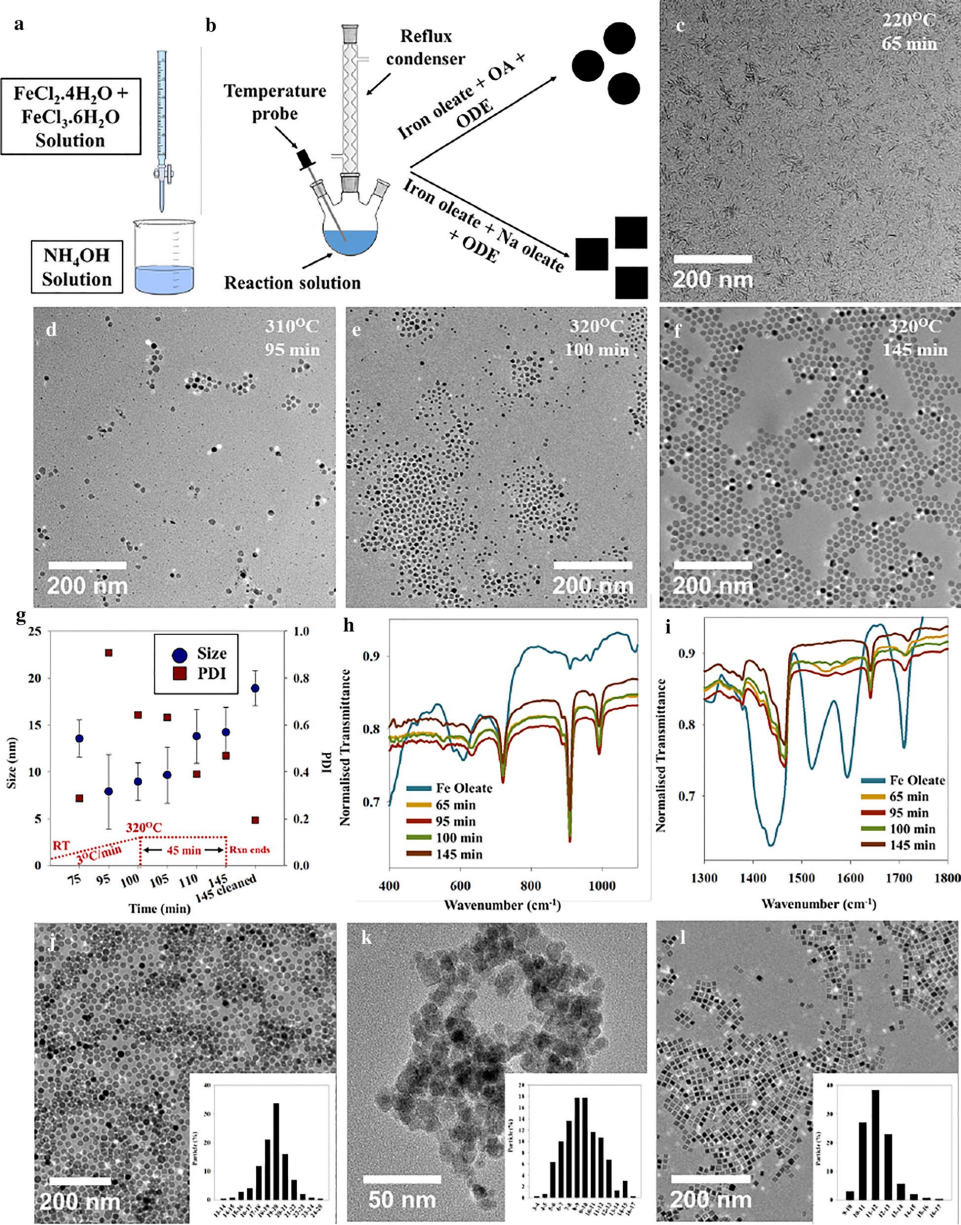
Schematic representing the synthesis route of IONPs via **a** co-precipitation and **b** thermal decomposition. **c**–**f** TEM images and **g** size/PDI, vs SNPs synthesis reaction times (RT = room temperature, Rxn = reaction). **h**, **i** FTIR spectra showing the development of SNPs with the decomposition of iron oleate. TEM images and size distribution histograms of the finally obtained IONPs **j** SNPs, **k** Mag and **l** CNPs

Figure 1a, b show the schematics of the synthesis routes of different IONPs via co-precipitation and thermal decomposition respectively. The ratio of the metal precursor (Iron oleate) to reducing/stabilizing (Na-oleate/OA ratio) agents influences the size, shape and morphology evolution of the IONPs. The growth of IONPs is governed by the decomposition of iron oleate precursor at high temperatures, which in turn influences the choice of solvent. On the other hand, the size of the NPs is clearly determined by the reaction time, temperature, and concentration [31]. TEM images in Fig. 1c–f, show the evolution of the spherical IONPs as a function of reaction time and reaction temperature. Nucleation starts at temperatures above 300 ℃ via ‘burst mechanism’ that ends quickly as the reaction enters growth regime and the NPs undergo sustained growth resulting in narrow particle size distribution as reported by others [32, 33]. However, we found the presence of some fully formed particles at 250 ℃ (Additional file 1: Figure S1a) that we attribute to stochastic growth arising from some premature decomposition of iron oleate resulting from heterogeneous nucleation at surface walls or via creation of pockets of supersaturation.

This burst nucleation along with the rapid increase in the number of particles can be distinctly seen from representative TEM images of the aliquot at the reaction temperature of 310 ℃ (Fig. 1d). Subsequently, the rapid consumption of the monomer causes the supersaturation to decrease and only the nuclei bigger than the critical size grow to form final NPs as the reaction time increases up to 320 ℃, after which the reaction is maintained for 45 min. (Fig. 1e, f) However, the supersaturation is high enough for diffusion-controlled growth that focuses on narrowing particle size distribution by allowing the smaller particles to grow rapidly compared to the bigger particles, owing to the higher surface energies of the smaller particles. The decrease in polydispersity along with the increase in mean particle size are characteristic of this growth regime and can be seen in Fig. 1g. Ostwald ripening may dominate in the later stages of the growth process that acts contrary to the diffusion-controlled growth as it allows bigger particles to grow on the expense of the smaller ones, thereby broadening the particle size distribution (PSD).

To further verify the mechanism discussed above, we have developed a model of particle formation which consists of an instantaneous reaction followed by nucleation and subsequent growth of nuclei (Additional file 1: Sect. 2). The growth consists of three main mechanisms, namely diffusion-controlled growth, coagulation due to Brownian collisions and Ostwald ripening. The simulation data matches well with the mean of the PSD from the experimental data of SNPs (Additional file 1: Figure S1b), however the standard deviation of the distribution is being overestimated from the model. We observe that the experimental data matches better (Additional file 1: Figure S1c) when Ostwald ripening is included in the mechanism. However, such a simplified model with several assumptions (viz. instantaneous reaction, lumped coagulation parameter, onset of Ostwald ripening) may not be adequate to capture the entire growth kinetics (Additional file 1: Sect. 4.1). For this reason, we leave the effect of Ostwald ripening on growth mechanism, as an open question that should be addressed through future experiments and simulations.

A monodisperse particle size distribution of SNPs is obtained as a result of distinct separation of the nucleation and growth regions resulting in final particle size of ~ 14 ± 3 nm [before cleaning (Fig. 1f)] and ~ 19 ± 2 nm [after cleaning (Fig. 1j)] [34]. The polydispersity of the particles is seen to decrease as NP size increases with the NPs becoming more spherical with reaction time and achieving a final PDI of ~ 0.2 (Fig. 1g). ImageJ software was used to calculate the particle sizes from the TEM images (i.e. Fig. 1c–f). 300–1000 particles were analysed per image and the average size and standard deviation of these have been plotted in Fig. 1g. Further, we investigated the growth mechanism by following the evolution of the FTIR spectra of the reaction aliquots taken out at specific time intervals as shown in Fig. 1h, i.

The bands around 1711 cm^−1^ can be attributed to the carbonyl group of the oleic acid (OA) whereas the vibrational frequencies at 1608, 1519, and 1444 cm^−1^ belong to carboxylate groups, characteristic of the oleate complex (Fig. 1i). In addition, the region between 1300 and 1700 cm^−1^ can be used to deduce the carboxylate coordination mode and the presence of these distinctive FTIR bands indicate that the structure of our oleate complex is bidentate as has also been reported by Bronstein et al. [33]. The removal of (OA) surrounding iron oleate starts around 170 ℃ and is followed by decomposition of the first oleate chain at 250 ℃, forming Fe^2+^ attached to two other oleate chains. Pure metal carboxylates decompose at temperatures near to or higher than 300 ℃ resulting in the formation of thermal free radicals [33]. The synthesis reaction for generating iron oxide nanocrystals is carried out by heating the solution of iron-oleate complex in a high boiling, long-chain hydrocarbon solvent, 1-octadecene (ODE). The gradual disappearance of the carboxylate peaks as a function of reaction time culminating in complete disappearance at end of reaction, as shown in Fig. 1i, stating successful decomposition of the metal-oleate complex, further supports our classical nucleation-growth model comprising burst nucleation and diffusion-controlled growth.

To gain information about the type of iron oxide formed in our system, FTIR of SNPs in the range 400–750 cm^−1^ were analysed (Fig. 1h). The absorption bands in the range 400–750 cm^−1^ are attributed to Fe–O vibration modes. IONPs can exist mainly in different polymorphs, magnetite (Fe_3_O_4_), hematite (α-Fe_2_O_3_), maghemite (γ-Fe_2_O_3_), as well as others designated as β-Fe_2_O_3_ which is rarely found. For Fe_3_O_4_, only one band exists around 574 cm^−1^ whereas, two and three bands can be seen for α-Fe_2_O_3_ and γ-Fe_2_O_3_ in the range 500–700 cm^−1^ respectively. From the XRD pattern (Additional file 1: Figure S1d), very broad features are observed confirming the small size of the NPs. Diffractions at 2*θ* values around 30°, 35°, 42° and 63° correspond to the lattice planes {220}, {311}, {400} and {440} respectively, of the cubic spinel z of magnetite [35, 36]. It is difficult to make a concrete distinction between the inverse cubic spinel magnetite (Fe_3_O_4_) and tetragonal maghemite (Fe_2_O_3_) phases from XRD due to their similar crystal structures. Furthermore, presence of maghemite could be attributed to oxidation on exposure to air during NP drying. The broad peak around 2*θ* = 20° could be due to OA, suggesting the coating of OA on NPs, acting as a stabilizing agent preventing agglomeration [36].

In the current study, besides the spherical IONPs discussed above (SNPs), two other IONPs were also synthesized, one using co-precipitation route (Mag) and other using the thermal decomposition route in the presence of sodium oleate (instead of OA) resulting in cubic IONPs (CNPs). Figure 1j–l, show representative TEM images of SNPs, Mag and CNPs respectively with 300 particles analysed for each respectively. Even though the co-precipitation route provides high product purity without unwanted organic solvents and use of high synthesis temperatures, this method produces NPs having a broader size distribution (without stabilizing agent). This is reflected by a higher poly dispersity index (PDI) of 0.24 in comparison to SNPs (PDI = 0.08) and CNPs (PDI = 0.09), synthesized via thermal decomposition methods. The higher degree of control of NP size in case of the latter method can primarily be attributed to temporal separation of nucleation and growth windows during the particle formation phase, leading to monodispersity. Further, thermal decomposition helps in controlling the shape of the IONP in the presence of stabilizing ligands (such as sodium oleate) as is reported here and in our previous studies [29]. Sodium oleate preferentially binds to the {100} facet over {111} preventing growth in that direction, allowing the {111} to grow faster leading to the formation of cube shaped NPs [37]. However, both SNPs and CNPs, being synthesized in organic solvents cannot be directly applied for hydrological applications which require the use of water dispersible tracers, thereby, necessitating phase transfer and subsequent functionalization of SNPs and CNPs.

### Phase Transfer and Functionalization of IONPs

#### Phase Transfer

In an attempt to transfer the SNPs and CNPs synthesized via thermal decomposition from organic solvent to water, functionalization strategies using various ligands are reported here. Table 2 shows the sizes and zeta potentials of the phase transferred SNPs and CNPs using different ligands. The phase transfer can primarily be achieved using two pathways; partial or complete replacement of the existing hydrophobic ligand with the hydrophilic ligand referred to as ‘ligand-ligand exchange’, or modification of the existing hydrophobic ligand with a hydrophilic ligand. The choice of ligands has been optimized through preliminary studies (not shown here) with an aim to control the surface charge of the phase-transferred NPs.

**Table 2:** DLS results showing the size and zeta potentials of the different phase transferred SNPs using various ligands

Iron precursor	Ligand	Size (nm)	Zeta potential (mV)
SNPs	Na-citrate	343 ± 5	− 49.7 ± 0.1
	CTAB	364 ± 10	32.0 ± 0.0
	TMAH	495 ± 11	− 10.0 ± 2.5
CNPs	TMAH	272 ± 20	− 38.6 ± 2.8

Both the NPs are seen to have hydrodynamic diameters, which are at least one order of magnitude higher than their respective TEM particle sizes (SNPs: ~ 19 ± 2 nm; CNPs: 12 ± 1 nm) before phase transfer. This suggests that during the process of functionalization, the ligand forms weak interactions among several NPs, resulting in larger hydrodynamic diameters. The reported functionalization strategies have been shown to be reproducible and robust (Additional file 1: Figure S2a and S2b), thereby allowing tunability of the surface functional groups, particle size and surface charge. Further, all the NPs show high electrostatic stabilities represented by high zeta potential values (positive or negative). The relative differences in zeta potentials among the samples reported can be traced back to different particle sizes, ligands, treatment conditions among others.

To further understand the surfaces of our phase transferred NPs and the functionalization mechanism, we performed a series of FTIR studies (Additional file 1: Figure S2c and S2d). Na-citrate functionalized SNPs are found to be anionic [38]. The sharp absorption bands at 1383 and 1584 cm^−1^ for pure Na-citrate show the stretching of asymmetric and symmetric carbonyl groups (C=O) respectively. These vibrations are greatly reduced for the Na-citrate functionalised SNPs showing interaction of C=O groups with the surface of SNPs. Concurrently, the absence of characteristic peaks of surface bound OA (stretching of asymmetric and symmetric hydrocarbon chains at 2850 and 2923 cm^−1^) in the case of SNPs with Na-citrate, further suggest complete replacement of OA. Therefore, ligand-ligand exchange happens whereby a small molecule (Na-citrate) easily displaces longer hydrocarbon chains that of surface bound OA, leading to negative surface charge in water. On the other hand, SNPs functionalized with CTAB are observed to be cationic. The absorbance bands for pure CTAB seen at 942 and 980 cm^−1^ depict C–N bond and –CH_3_ groups stretching respectively (Additional file 1: Figure S2c and S2d). For SNPs coated with CTAB, the vibrations at 942 and 980 cm^−1^ are highly reduced and shift in comparison to CTAB. This is due to the interaction of the N^+^ group with SNPs leading to reduced absorbance of its own C–N bond stretching, confirming SNPs’ functionalization with CTAB. Although the intensity of the surface bound OA bands at 2850 and 2923 cm^−1^ is reduced, these overlap with CTAB hydrocarbon bands in the same range. Therefore, we believe that chemisorption of CTAB molecules on the SNP surface happens either via complete removal of the surface bound OA or formation of inclusion complexes with OA, characteristic of a quaternary amine, reported in case of other quaternary amines such as α-cyclodextrin [38].

**Fig. 2:**
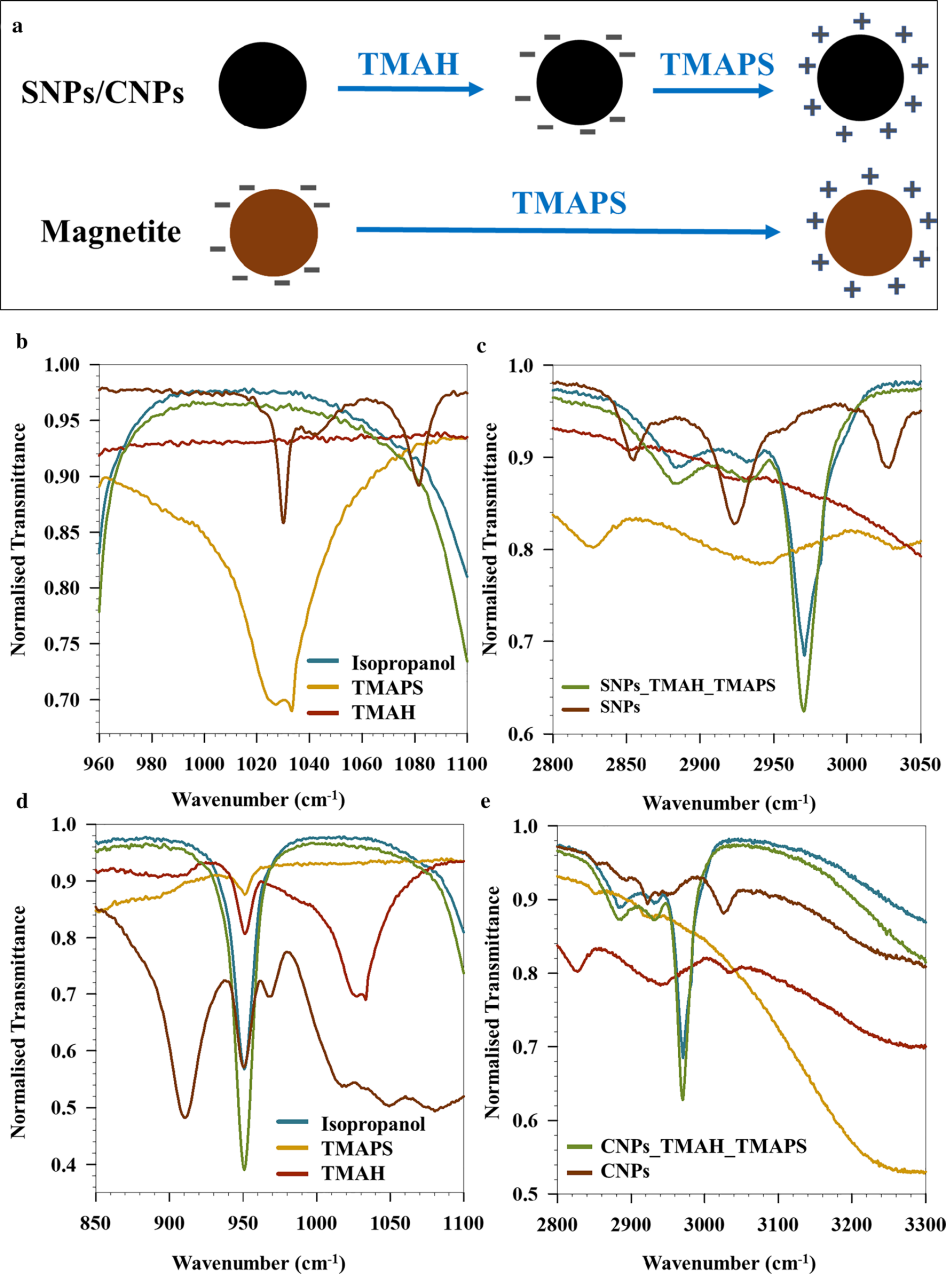
**a** Schematic representing the surface functionalization of different IONPs with TMAH and TMAPs. FTIR spectra of the surface functionalized **b**, **c** SNPs and **d**, **e** CNPs with TMAH and TMAPS

Unlike the CTAB functionalized SNPs, for successful binding of negatively charged DNA to the surface of phase transferred IONPs, it is imperative to decorate the surface with positive charges. This is achieved by first phase transferring SNPs and CNPs with TMAH (as described above), and subsequently functionalizing with TMAPS. On the other hand, this phase transfer protocol is not needed for Mag NPs (synthesized via co-precipitation) which are directly functionalized with TMAPS. A schematic representation is shown in Fig. 2a.

#### Surface Charge Modification

**Fig. 3:**
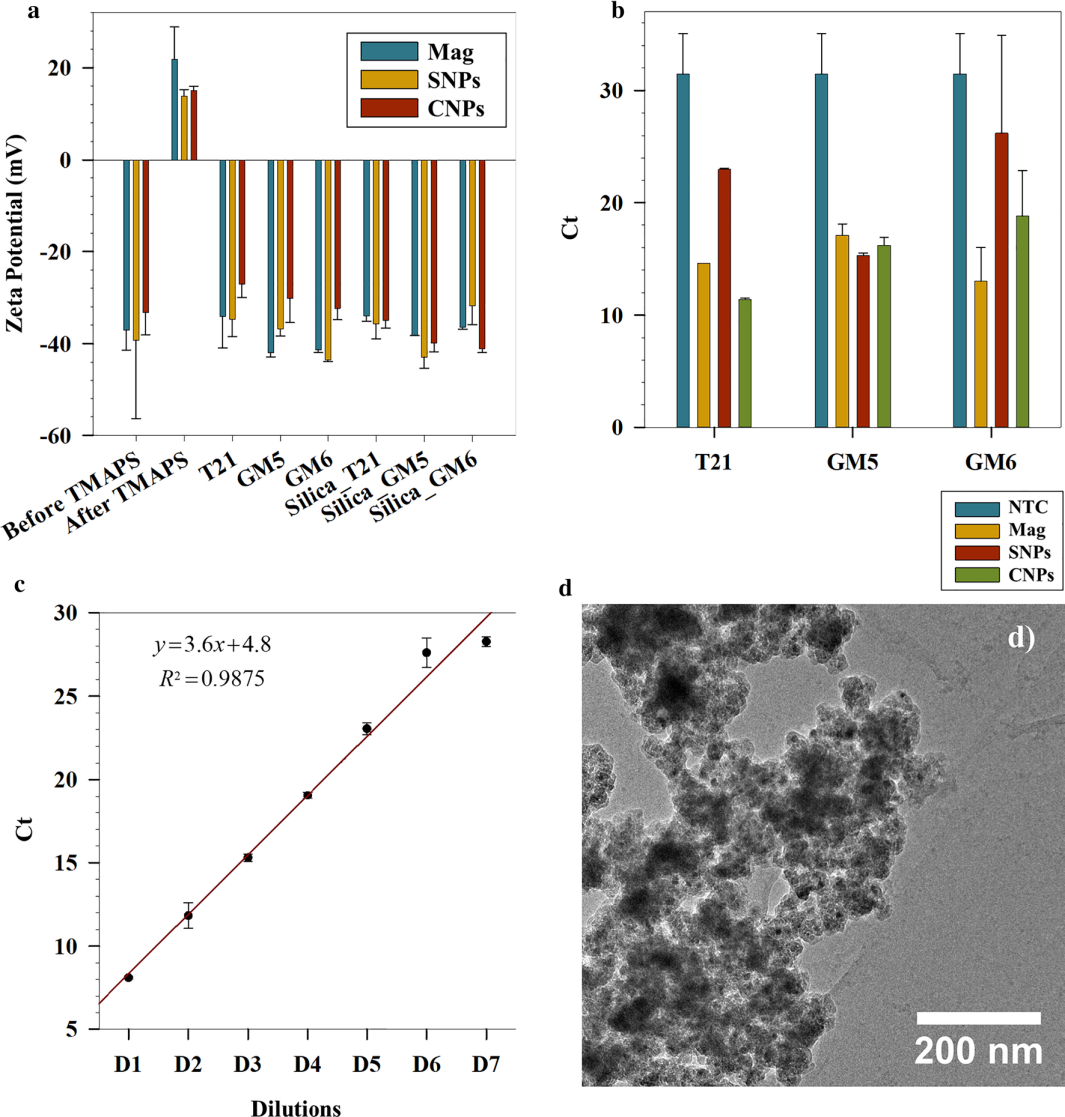
**a** Zeta potential values of the IONPs after different surface functionalization steps. **b** qPCR cycles for the different NPs functionalized with different dsDNA. **c** Dilution curve for GM5 functionalized SNPs up to 7 dilution cycles. **d** TEM image of T21 functionalized Mag NPs encapsulated in silica. (The error bars represent the standard deviation calculated from the triplicate measurement of the particular sample)

Figure 2b shows the ATR-FTIR spectra obtained for studying the surface modification of SNPs and CNPs functionalized with THAH and further with TMAPS. The absorbance at 1031 cm^−1^ for TMAPS corresponds to the stretching of –Si–O–C– [39]. This vibration is absent in case of both SNPs and CNPs functionalized with TMAH and TMAPS (Fig. 2b, c) owing to the interaction of –N(CH)_3_ group of TMAH with –Si–O– of TMAPS during the modification process. Complete removal of OA can be concluded from the disappearance of the asymmetric and symmetric long chain hydrocarbon vibrations of OA at 2850 and 2923 cm^−1^ (Fig. 2b). Appearance of a sharp absorbance band at 2972 cm^−1^ in case of SNPs functionalized with TMAH and TMAPS (shown in Fig. 2b) is due to singly bonded –C–H groups of isopropanol, TMAPS and TMAH. The sharp peak seen at 920 cm^−1^ for CNPs (Fig. 2d) can be attributed to out-of-plane angular deformation of –C–OH of Na-oleate (present on the surface of CNPs) [40]. Complete disappearance of this peak is seen for spectra of CNPs functionalized with TMAH and TMAPS showing removal of Na-oleate which is in sync with phase transferred SNPs (where OA was removed). Other spectral features at 1031 (Fig. 2d), 2876 and 2972 cm^−1^ (Fig. 2e) for CNPs functionalized with TMAH and TMAPS have similar characteristics as of SNPs functionalized with TMAH and TMAPS, showing analogous surface interactions during phase transfer. From the FTIR data, it is however difficult to conclude if TMAPS replaces TMAH completely for either SNPs or CNPs. However, it can be inferred that TMAPS is the outermost ligand providing positive zeta potential after TMAPS coating in case of Mag, SNPs and CNPs as shown in Fig. 3a, arising from the amine functional group of TMAPS.

#### Silanization and Binding of DNA

After successful functionalization of the particles yielding in cationic surfaces, the three NPs viz. Mag, SNPs and CNPs were loaded with dsDNA. Figure 3a shows the variation in zeta potentials at each surface functionalization step for all the IONPs. TMAPS was successfully bound to the respective NP surface, represented by positive zeta potentials after TMAPS functionalization (Mag: 21 ± 7 mV, SNPs: 13 ± 1 mV, CNPs: 15 ± 1 mV).

With an intent to fabricate tracers, bar-coded with unique dsDNA, we chose three different dsDNA molecules, namely, T21, GM5 and GM6. Charge density mapping between the TMAPS functionalized IONPs and the dsDNA is a precursor to the successful binding of the dsDNA to the IONPs. Through a series of preliminary experiments, we optimized the ratio of NPs to dsDNA to facilitate effective binding. Successful binding of DNA reverses the surface charge of the IONPs in all the cases as reported in Fig. 3a. This shows the robustness and adaptability of our protocol to different particle types and different DNA strands. A final coating of silica was carried out after this step to ensure a protective shell around the DNA. The silica coated NPs containing specific dsDNA and specific iron oxide core are all found to have high colloidal stabilities indicated by high zeta potential values (Fig. 3a).

In order to assess the dsDNA loads in our tracer particles, qPCR measurements were done after bleaching any surface bound dsDNA. To release the encapsulated DNA, the tracer particles were dissolved in buffered oxide etch—a fluoride buffer, containing (NH_4_)HF_2_ and NH_4_F, which has been shown to etch away the silica (shell) and iron oxide (core), without affecting the dsDNA [14]. In addition, the presence of this buffer in the qPCR samples has been previously shown neither to influence the signal to noise ratio nor inhibit the primers during amplification cycles [14]. The cycle threshold (Ct) values obtained from qPCR runs for the respective tracer particles are shown in Fig. 3b. A lower C_t_ value indicates that the fluorescent signal required to cross the threshold (exceed background level) is defined by a smaller number of cycles, in turn representing high DNA concentrations. The presence of large detectable quantities of DNA is therefore verified for all the tracer types, indicated by statistically significant lower C_t_ values compared to NTC (negative control). The results shown are for D3 diluted samples (tenfold dilution with each cycle), whereby further proving the applicability of such tracers with high ultra-sensitivity and negligible dilution problems. To further substantiate our claim, we performed a dilution series for the SNP tracers functionalized with GM5 as shown in Fig. 3c. The PCR efficiency is determined by means of a standard curve involving generating a dilution series of the concentrated stock solution. This series of samples, with controlled relative amounts of targeted template, is most frequently diluted using tenfold dilution steps that are analysed by qPCR measuring the quantification cycle (C_t_) using standard procedures. An efficiency of 100% follows the assumption of perfect doubling of the number of DNA template molecules in each step of the PCR. The PCR efficiency (*E*) can be calculated from the slope of the C_t_ versus the logarithm of the target concentrations as follows [41]:

1 $$E = ({10^{(1/{\text{slope}})}} - 1) \times 100\%$$

2 $$E = ({10^{(1/3.6)}} - 1) \times 100\% = 89.6\%$$

with $${R^2} = 0.9875.$$

Also, D6 and D7 show similar C_t_ values that are close to the NTC of the assay. Hence, there could still be some particles left in D7, however, the C_t_ value is obscured by the NTC.

Figure 3d shows a representative TEM image of T21 functionalized Mag tracers, Although, several Mag NPs can be distinctively seen throughout a mesh of silica, our results show that several Mag NPs are encapsulated within single silica shells. However, how sequential functionalization affects the magnetic properties of IONPs needs to be studied in order to assess their applications in tracer hydrology and subsequent magnetic separation prior to analysis. We report here for the first time an in-detail magnetic characterization of our magnetic DNA tracers at each stage of functionalization.

### Influence of Magnetic Properties upon Functionalization

Molecular coating can strongly influence magnetic properties of NPs both restoring the bulk saturation magnetization and influencing the local effective magnetic anisotropy, (i.e. coercive field). This effect can be attributed to the influence of different ligands bonded at the IONPs surface that modify electronic structure and then magnetic properties of the NPs [26, 27, 42]. In order to investigate the effect of molecular coating on magnetic properties, field and temperature dependence of magnetization has been investigated on bare magnetite (Mag) and at every stage of functionalization (Mag_TMAPS, Mag_DNA and Mag_Silica) particles. Even though SNPs and CNPs possess higher structural uniformity that might lead to superior magnetic properties, we limit our discussion of magnetic measurements to Mag, as these NPs provide higher saturation magnetization with respect to CNPs (*M*_s_ = 39 (3) Am^2^ kg^−1^) in our case (Additional file 1: Figure S5). In addition, Mag provides the most consistent qPCR results (Fig. 3b) across the 3 variants of dsDNA used. Furthermore, studies conducted on Mag have not shown any magnetic data to stress on the effect of surface functionalization and encapsulation on the magnetic properties of these NPs.

**Fig. 4:**
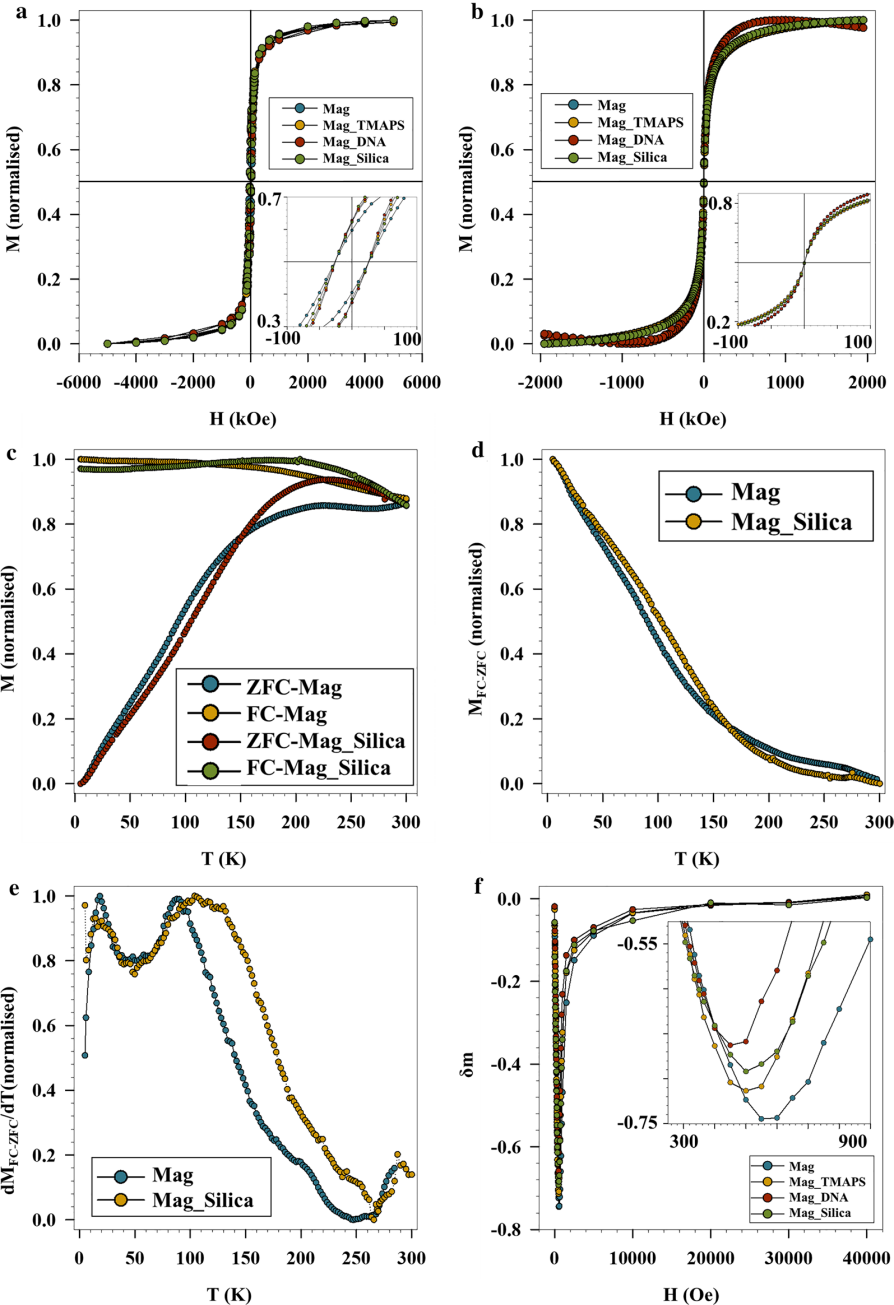
Field dependence of magnetization for Mag, Mag_TMAPS, Mag_DNA and Mag_Silica at **a** 5 K and **b** 300 K. **c** ZFC–FC measurements, **d** *M*_FC_–*M*_ZFC_ plots and **e** derivative of *M*_FC_–*M*_ZFC_ plots for Mag and Mag_Silica. **f** *δm* plots at 5 K Mag and functionalized Mag samples

Field dependence of magnetization recorded at 5 K (Fig. 4a) indicates that the coating of Mag was successfully achieved without affecting the magnetic characteristics of the magnetic core and both bare sample and coated samples present very close saturation magnetization (*M*_s_) values, and coercivity field, *H*_c_ (inset Fig. 4a, Table 3). *M* vs *H* at 300 K (Fig. 4b) shows superparamagnetic behaviour (i.e. Mr = 0 and Hc = 0) for all the samples. Thermal dependence of magnetization measured according to zero field cooled (ZFC) and field cooled (FC) protocols in DC field of 25 Oe and in the temperature range of 5–300 K are shown in Fig. 4c. All the coated samples show comparable magnetic behaviour that are different from bare NP counterparts. In this view, in order to draw a clearer picture, a comparison among Mag and Mag_Silica particles will be shown as an example and ZFC FC measurements for all the samples is reported in Additional file 1: (Sect. 4.3). According to ZFC protocol, the sample was at first cooled down from 300 to 5 K in zero field, then a small field of 25 Oe was applied and the data was collected during the warming up from 5 to 300 K. The applied field was maintained as the sample was cooled down again to 5 K and M_FC_ magnetization was measured during the cooling process. As an example, Fig. 4c shows the ZFC–FC of Mag and Mag_Silica samples. ZFC–FC exhibit a blocking process typical of an ensemble of single-domain magnetic particles with a distribution of blocking temperatures [43]. Both MZFC of Mag and Mag_Silica sample clearly show a maximum that can be considered proportional to the mean blocking temperature:

3 $${T_{\max }} = \beta {T_B}$$

**Table 3:** Coercive Field (H_c_), saturation magnetization at 5 K (M_S_). Temperature corresponding to the maximum in ZFC curve (*T*_max_), irreversibility temperature (*T*_irr_), and blocking temperature (*T*_B_). Uncertainties on the last digit are given in parentheses

Sample	Hc (Oe)	*M*s 5 K (emu/g)	*T*_max_ (K)	*T*_B_ (K)	*T*_irr_ (3%) (K)
Mag	260 (25)	77 (8)	228 (22)	90 (9)	283 (28)
Mag_TMAPS	255 (20)	75 (8)	236 (23)	92 (9)	243 (24)
Mag_DNA	250 (20)	85 (9)	202 (20)	92 (9)	207 (20)
Mag_Silica	200 (20)	83 (9)	228 (22)	102 (10)	242 (24)

where *β* can be considered in the range 1.5–2.5 for log normal size distribution [44].

Both samples show also irreversibility between FC and ZFC up to quite high temperature. The temperature below this irreversibility is observed (*T*_irr_) and can be associated with the blocking of the biggest particles. Looking at values of *T*_max_ and *T*_irr_ (Table 2), it appears clear that all the samples show similar values within experimental errors.

It is worth to note that *M*_FC_ shows temperature independent behaviour at low temperature, suggesting the presence of relevant interparticle interaction among the particles [45].

In order to better understand the effect of the molecular coating on the magnetization dynamics of the NPs, the temperature dependence of the difference *M*_FC_ − *M*_ZFC_ has been plotted (Fig. 4d) (*M*_FC_ − *M*_ZFC_ of the other samples are reported in Additional file 1: Figure S3a). For a NP ensemble, it can be demonstrated that

4 $${M_{{\mathrm{TRM}}}}(H,T,t) = {M_{{\mathrm{FC}}}}(H,T,t) - {M_{{\mathrm{ZFC}}}}(H,T,t) + {M_{{\mathrm{IRM}}}}(H,T,t)$$

where *M*_IRM_ is isothermal remnant magnetization. However, as *M*_IRM_ is negligible in the IONP assemble, *M*_FC_ − *M*_ZFC_ can be considered as a good approximation of *M*_TRM_ [46, 47]. For both samples, *M*_FC_ − *M*_ZFC_ shows a decrease with increasing temperature, as expected for an assembly of magnetic monodomain particles. The derivative of *M*_FC_ − *M*_ZFC_ (Fig. 4e) can be considered as only a rough estimation of the Δ*E*_a_ distribution due to the presence of interparticle interactions in our samples. The distribution of magnetic anisotropy energies shows the presence of two maxima centered at around 20 K (*T*_Low_) and 100 K (*T*_high_) respectively. Following previous detailed investigation of magnetic properties in iron oxides, *T*_low_ can be ascribed to the freezing of non-collinear spin present in the particle surface, while *T*_high_ is related to superparamagnetic transition of monodomain particles [48–50].

Within the Néel model, the blocking temperature can be defined as the temperature for which the relaxation time is equal to the measuring time of the experimental technique. In a real system of NPs, where a finite size distribution always exists, *T*_B_ is often defined as the temperature at which 50% of the sample is in the superparamagnetic state. The *T*_B_ distribution can be obtained from the Δ*E*_a_ distribution by evaluating the temperature at which 50% of the particles overcome their anisotropy energy barriers. Blocking temperature for all the samples show equal values within experimental errors, suggesting that molecular coating is not influencing the magnetization dynamics of superspin. It is interesting to observe that data reported in Table 3 is in good agreement with Eq. 3: considering the values of *T*_max_ and *T*_irr_, *β* are in the expected range. This suggests that despite interparticle interaction being present between particles, the magnetization dynamics of superspin is governed by magnetic anisotropy of single particles [43, 51].

In order to shed some light on the interparticle interactions in our samples, the variation of δm versus field at 5 K according to DCD (Direct Current Demagnetization) and IRM (Isothermal Remnant Magnetization) protocols (Additional file 1: Sect. 4.3), are presented in Fig. 4f [52]. As an example, positive value in δm is an indication of exchange interactions among nanoparticles while negative peak indicates the prevalence of dipolar interactions. Thus, it can be clearly seen from Fig. 4f, that the dipolar interaction is dominating in our samples, before coating as well. After coating with DNA, a slight, but evident, reduction in the intensity of δm (as absolute value) was observed (inset, Fig. 4f). In a sample of randomly distributed nanoparticles with average magnetic moment *μ*_p_ (i.e. *M*_s_ × *V*_p_,) and average separation *d*, dipolar energy (*E*_d_) is approximately, given by

5 $$E_{{\text{d}}} \;\sim\;\frac{{\mu _{0} (M_{{\text{s}}} \times V_{{\text{p}}} )^{2} }}{{4\pi d^{3} }}$$

where *μ*_0_ is permeability, *M*_s_ saturation magnetization, *V*_p_ volume of nanoparticles, *d* is the distance between particles. Considering *M*_s_ and *V*_p_ equal in all the samples, the observed reduction of dipolar interactions (i.e. dipolar energy) means an increase of interparticle distance, indicating the efficiency for the coating process.

The magnetic properties (i.e. saturation magnetization and magnetic anisotropy) of Mag remain intact after surface functionalization with DNA and encapsulation in silica proving the efficiency of magnetic core and the possibility of magnetic separation of these NPs thereby improving the recovery of the tracer moieties downstream.

## Conclusion

Conventional hydrological tracers show limitations due to lack of multiplexed, multipoint tracing and interference of background noise. DNA based tracers eliminate most of these challenges by enabling synthesis of ideally unlimited number of unique tracers that are stable and highly sensitive to detection. Herein, we synthesised DNA based tracers having a magnetic core encapsulated in a protective silica shell. We report for the first time encapsulation of three different types of DNA using three different magnetic cores, thereby showing the uniqueness of the tracers. Superparamagnetic IONPs, with a size range of 10–20 nm, were synthesized using the methods of co-precipitation and thermal decomposition for the purpose of producing magnetic hydrological tracers. The surface of the IONPs were functionalized using different ligands such as CTAB, Na-citrate, TMAH and TMAPS, thus, providing different surface functionalities and surface charges to IONPs. The functionalized nanoparticles show high stability denoted by high zeta potential values, above > 30 mV (CTAB) and < − 30 mV (Na-citrate and TMAH). Spherical and cube shaped IONPs, synthesized via co-precipitation or thermal decomposition routes, were thereafter successfully functionalized with three different dsDNA (T21, GM5 and GM6) followed by encapsulation in silica shell. The effect of surface functionalization of IONPs on the physico-chemical properties and especially magnetic properties of IONPs was investigated. In the latter context, we track for the first time, the effect of functionalization on the magnetic properties. It was observed that the magnetic core retained most of its magnetic properties even after encapsulation in silica. Successful DNA binding to the IONPs was verified using qPCR for all the different magnetic cores and the three different dsDNAs studied herein. Our results show robust and repeatable synthesis and functionalization protocols capable of producing magnetic tracers with unique DNA tags that retain relevant magnetic properties of the cores even after silica encapsulation. The tracers show detection at extremely low concentrations, thereby proving their non-susceptibility to dilution effects under application conditions. These tracers will pave the path for monitoring several hydrological processes, currently limited by the availability of multiple unique tracers. Further, the use of DNA as a tag is a precursor to ultra-high sensitivity in detection and the magnetic core will facilitate easy separation of tracers after applications downstream.

## Supplementary Information

**Additional file 1:** Supporting information.

## Data Availability

All additional and relevant data can be found with the Supporting Information.

## Abbreviations

qPCR: Quantitative polymerase chain reaction
PAA: Polyamidoamine
PEG: Poly(ethylene) glycol
NPs: Nanoparticles
IONPs: Iron oxide nanoparticles
PLA: Polylactic acid
TMAH: Tetra methyl ammonium hydroxide
CTAB: Hexadecyltrimethylammonium bromide
ODE: 1-Octadecene
OA: Oleic acid
TEOS: Tetraethyl orthosilicate
TMAPS: Trimethoxysilylpropyl-*N,N,N*-trimethylammonium chloride
Mag: Magnetite NPs synthesised by co-precipitation
SNPs: Iron oleate: spheres
CNPs: Iron oleate: cubes
TEM: Transmission electron microscopy
FTIR: Fourier transform infrared
PSD: Particle size distribution
DLS: Dynamic light scattering
SQUID: Super conducting quantum interference device
XRD: X-ray diffraction
ZFC–FC: Zero field cooled–field cooled
BOE: Buffered oxide etch
