# Supplementary material for: Magnetic Nanoparticles to Unique DNA Tracers: Effect of Functionalization on Physico-chemical Properties
Source: Nanoscale Res Lett. 2021 Feb 6;16:24. doi: 10.1186/s11671-021-03483-5 (PMC7867676; doi:10.1186/s11671-021-03483-5)
Supplement: Supplementary file 1 — Additional file 1: Supporting information. [file 11671_2021_3483_MOESM1_ESM.docx]

Supporting Information

Magnetic Nanoparticles to Unique DNA Tracers – Effect of Functionalization on Physico-Chemical Properties.

Anuvansh Sharma^1^, Jan Willem Foppen^2^, Abhishek Banerjee^1^, Slimani Sawssen^3,4^, Nirmalya Bachhar^5^, Davide Peddis^3,4^, Sulalit Bandyopadhyay^6,7*^

1 Department of Materials Science and Engineering, NTNU Norwegian University of Science and technology, N-7491 Trondheim, Norway.

2 Water Science and Engineering, IHE Delft Institute for Water Education, Delft, Netherlands.

3 Dipartimento di Chimica e Chimica Industriale, Università di Genova, Genova, Italy

4 Istituto di Struttura della Materia – CNR, Area della Ricerca di Roma1, Monterondo Scalo, RM 00015, Italy

5 Department of Chemical Engineering, Indian Institute of Technology Jodhpur, Jodhpur 342037, India.

6 Department of Water Management, Delft University of Technology (TU Delft), Netherlands.

7 Department of Chemical Engineering, NTNU Norwegian University of Science and technology, N-7491 Trondheim, Norway.

* Correspondence: sulalit.bandyopadhyay@ntnu.no Tel.: +47-45071041

*1. Materials and Methods*

The following dsDNA strands were used for the studies.

*1.1. T21*

Sense: 5'–*CTG GAT GAT GCG AAT CGA ATC ACA CAC GTA GAG TTC GAA TGT GAT GGC CTA AAA GGA TGA GCA ATA GAG GAG CCT AAA GG–3'*

Antisense: 5'–*CCT TTA GGC TCC TCT ATT GCT CAT CCT TTT AGG CCA TCA CAT TCG AAC TCT ACG TGT GTG ATT CGA TTC GCA TCA TCC AG–3'*

*1.2. GM5*

Sense: *5'–TTC GGA CAA TCC TTT CCA TAT TAC GCT CTG AAG GCT ACT ACT CCT TCT TAT TAA CTG GGT CTC GTT T–3'*

Antisense: *5'–AAA CGA GAC CCA GTT AAT AAG AAG GAG TAG TAG CCT TCA GAG CGT AAT ATG GAA AGG ATT GTC CGA A–3'*

*1.3. GM6*

Sense: *5'–TTC TCT GCC CTT ACG TTT ATC TTA AGG GCC GGT CCA CCA GTT GAA CAC GAA CAA ACC TCT TT–3'*

Antisense: *5'–AAA GAG GTT TGT TCG TGT TCA ACT GGT GGA CCG GCC CTT AAG ATA AAC GTA AGG GCA GAG AA–3'*

*2. Model and simulation of particle formation and growth*

*2.1. Nucleation*

In our model, we assume homogeneous nucleation to take place during thermal decomposition and the nucleation frequency, (*f*_n_), can be calculated by classical nucleation theory as:

$f_{\text{n}}=\left\{\begin{matrix} 0\text{ if, }\lambda_{l}<1 \\ VA\text{ }\exp\left(\frac{-\text{ }16\pi\sigma^{3}V_{m}^{2}}{3(k_{B}T)^{3}\text{ }\ln(\lambda_{l})^{2}}\right)\text{ if, }\lambda_{l}\geq1 \end{matrix}\right.$ (S1)

where, *λ_l_* is the degree of supersaturation, defined by:

$\lambda_{l}=\frac{C(l)}{S}$ (S2)

and *V, A*, *σ*, *k*_B_, *T*, *S*, *C*(*l*) and *V*_m_ are total volume of liquid solution, pre-exponential factor, interfacial tension between solid nuclei and the surrounding liquid, Boltzmann constant, temperature, solubility of product molecule (Fe_3_O_4_), concentration of Fe_3_O_4_ in liquid phase and Fe_3_O_4_ molecular volume, respectively.^[1]^ The values of all model and experimental parameters are given in **Table S1**. For critical nuclei size we considered the smallest possible cluster (*l*_c_ = 2 molecules, the smallest cluster) in our simulation, since a critical number of 2 is often used for nucleation of a sparingly soluble product.^[2-4]^

*2.2. Diffusion controlled growth*

The growth of nanoparticles by diffusion transport of molecules to its surface consists of two steps: (i) diffusion of solute molecule through a boundary layer (diffusion layer) from the bulk to the solid surface and (ii) surface-reaction of solute molecules that bind it to the nanoparticle. Generally, surface-reaction of molecules on the particle surface are very fast, therefore we ignore this step in our model. Hence, diffusion transport driven growth rate of iron oxide nanoparticles can be written as,

$\frac{dr}{dt}=\frac{D_{m}V_{m}N_{A}C(l)}{r}$ (S3)

where *D*_m_, *N_A_* and *r,* are molecular diffusivity of solute molecule, Avogadro’s number and radius of particle, respectively.^[5, 6]^ Diffusivity of Fe_3_O_4_ molecules in octadecene has been estimated by the Wilke-Chang correlation for molecular diffusion.^[7]^

*2.3. Coagulation*

Brownian collisions of the nanoparticles which result in complete coalescence (coagulation), leading to primary particle growth. The coagulation driven growth rate of a nanoparticle is therefore estimated using the Brownian collision frequency (*q*_p_) modified by a coagulation efficiency, *β*.^[1]^ The efficiency *β* is the ratio of the number of successful Brownian collisions (leading to coagulation), against the total number of Brownian collisions. *q*_p_ is given by Smoluchowski’s equation:

$q_{p}=\frac{2k_{B}T}{3\mu}\left[2+\frac{r_{1}}{r_{2}}+\frac{r_{2}}{r_{1}}\right]$ (S4)

where *r*_1_ and *r*_2_ are radii of colliding particles and *μ* is viscosity of the solvent.^[8, 9]^ The coagulation frequency is hence calculated as:

$f_{c}=\frac{1}{2}\beta Vq_{p}N^{2}$ (S5)

where *N* is the number density of nanoparticles.

*2.4. Ostwald ripening*

The timescale of Ostwald Ripening (OR) is large compared to all other formation and growth processes. The rate constant of OR, *k*_OR_ can be written as,

$k_{OR}=\frac{8\sigma D_{m}(V_{m}N_{A})^{2}S}{9RT}$ (S6)

where *R* is universal gas constant (8.314 JK^-1^mol^-1^). Oskam *et al.* have shown by linearizing the Gibbs-Thomson equation, that the condition $\frac{4\sigma V_{m}}{k_{B}T}<<\overline{d}_{p}$ must be valid, to have Ostwald ripening take place in a system.^[10]^ Based on our parameter values of Fe_3_O_4_ nanoparticle, $2\alpha=\frac{4\sigma V_{m}}{k_{B}T}=12$nm. Therefore, we assume the onset point of OR in our system is 12 nm. The rate of the corresponding coarsening process can be given as:

$\frac{dr}{dt}=\frac{3\alpha D_{m}V_{m}N_{A}S}{r^{2}}\left(\frac{r}{r_{c}}-1\right)$ (S7)

where *dr*/*dt* represents the growth or decay in particle radius depending on whether *r*/*r*_c_ is greater or less than 1, respectively.^[11]^ The rate equation of OR implies that in the PSD, particles larger than *r*_c_ will grow while smaller particles dissolve. Here critical radius *r*_c_ is given by, *r*_c_ = *α* /*θ*(*t*), where, *θ*(*t*) = [*C*(*l*)-*S*]/*S*, is a dimensionless concentration.^[11]^ We have arbitrarily fitted *θ*(*t*) to be 1 to match the PSD.

**Table S1**. List of input parameters in the simulation.

| **Parameter** | **Value** | **Source** |
| --- | --- | --- |
| *C*(*l*)*_t_*_=0_ | 23.7 mol m^-3^ | our experiment |
| *V* | 2.5 × 10^-5^ m^3^ | our experiment |
| *V*_b_ | 2.8×10^-19^ m^3^ | our simulation |
| *T* | 593 K | our experiment |
| *μ* | 0.3 kg.m^-1^s^-1^ | obtained from literature |
| *D*_m_ | 2.28 × 10^-8^ m^2^s^-1^ | Estimated using Wilke-Chang correlation ^[7]^ |
| *l*_c_ | 2 | obtained from literature ^[4]^ |
| *A* | 10^34^ m^-3^s^-1^ | obtained from literature ^[4]^ |
| *σ* | 0.1 J.m^-2^ | obtained from literature ^[4]^ |
| *V*_m_ | 7.39 × 10^−29^ m^3^ | calculated using molar mass, density, and Avogadro Number |
| *S* | 10^-6^ mol m^-3^ | - |
| *β* | 2.5 × 10^−5^ | Our simulation |
| *r*_c_ | 6 nm | Our simulation |
| *θ*(*t*) | 1 | Our simulation |

*2.5. Simulation methodology*

We formulated our kinetic Monte Carlo (kMC) simulation following Bachhar *et al*., by adopting the technique of interval of quiescence.^[5, 6]^ There are four events in the simulation, namely, nucleation, diffusion-growth, coagulation-growth and Ostwald ripening. The interval of quiescence is defined as the time interval between two consecutive, discrete random events (nucleation or coagulation in the present case). During this interval, other continuous events (diffusion-growth and Ostwald ripening, estimated by their respective rate equations) occur. We start our simulation using a simplified growth model, with *n*_m_ number of Fe_3_O_4_ molecules (which at *t* = 0 is 4×10^6^) and no nanoparticle to start with (*N*_p_ = 0). We used a discrete–continuous kMC simulation following Bachhar *et al.* ^[5, 6]^

3*. Phase transfer of SNPs*

*3.1. CTAB*

The method has been adapted and modified by previous work of Kim et al. and 10 mM of CTAB solutions were made in 20 mL MilliQ water.^[12]^ 5 and 10 mg of SNPs synthesized from thermal decomposition route were separately precipitated from toluene using methanol and cleaned thrice with acetone. Each precipitated SNP sample was redispersed in 2 mL chloroform before adding it dropwise to 20 mL of 1 and 10 mM CTAB solutions preheated to 60 ℃ over an oil bath under constant stirring. The reaction was left for 30 min after which the phase transferred SNPs were magnetically separated and washed thrice with MilliQ water before suspending them in 5 mL MilliQ water.

*3.2. Sodium citrate*

The procedure is taken and modified from works of Bandyopadhyay et al.^[13]^ 10 and 50 mM of sodium citrate (Na-citrate) solutions were prepared in 10 mL MilliQ water. 5 and 10 mg SNPs were separately precipitated from toluene using methanol and cleaned thrice with acetone. 10 ml Na-citrate solution was added to the precipitated SNPs and the suspension was sonicated for 2 hours in a bath sonicator. Afterwards, the nanoparticles were magnetically separated and cleaned thrice with MilliQ water before redispersing them in 10 mL MilliQ water.

*3.3. TMAH*

Phase transfer of SNPs and CNPs using TMAH was performed by modifying a previously developed method.^[14]^ Typically, 50 mg of SNPs and 25 mg of CNPs were separately precipitated from toluene using methanol and washed thrice with acetone in a glass vial. 2 mL and 1 mL TMAH solution was added to the SNPs and CNPs respectively and the vials were placed in a bath sonicator for 10 minutes. Afterwards, the vials were put on shaking at 300 rpm for 2 hours after which the particles were magnetically separated and cleaned thrice with water. Finally, the phase transferred IONPs were redispersed in 1 mL MilliQ water in an Eppendorf tube.

**Table S2**. SNPs from different precursors with different ligands

| SNPs precursor | SNPs mass (mg) | Ligands | Ligand concentration (mM) |
| --- | --- | --- | --- |
| Iron oleate: SNPs spheres | 5 | CTAB | 1 |
|  | 10 |  | 1 |
|  | 5 |  | 10 |
|  | 10 |  | 10 |
| Iron Oleate: SNPs spheres | 5 | Sodium citrate | 10 |
|  | 10 |  | 10 |
|  | 5 |  | 50 |
|  | 10 |  | 50 |

*4. Results and discussion*

*4.1. Controlling the Particle Size and Shape*

Based on the TEM analysis an experimental PSD has been constructed as reported in **Figures S1a and S1b**, where the relative frequency (%) of each size are plotted against particle diameter. Our model can capture the mean and the mode of the PSD however the standard deviation and other higher moments of the distributions are not accurately captured. A much more detailed model with OR might need to be considered for this process. The only additional adjustable parameter in this simulation was the coagulation efficiency which we found to be 2.5 × 10^−5^. It is observed that the simulation data matches well with OR included. In this case the coagulation efficiency is found to be 8.5 × 10^−6^. A detailed model of coagulation based on interparticle interaction^6^ might give us better understanding of the process.


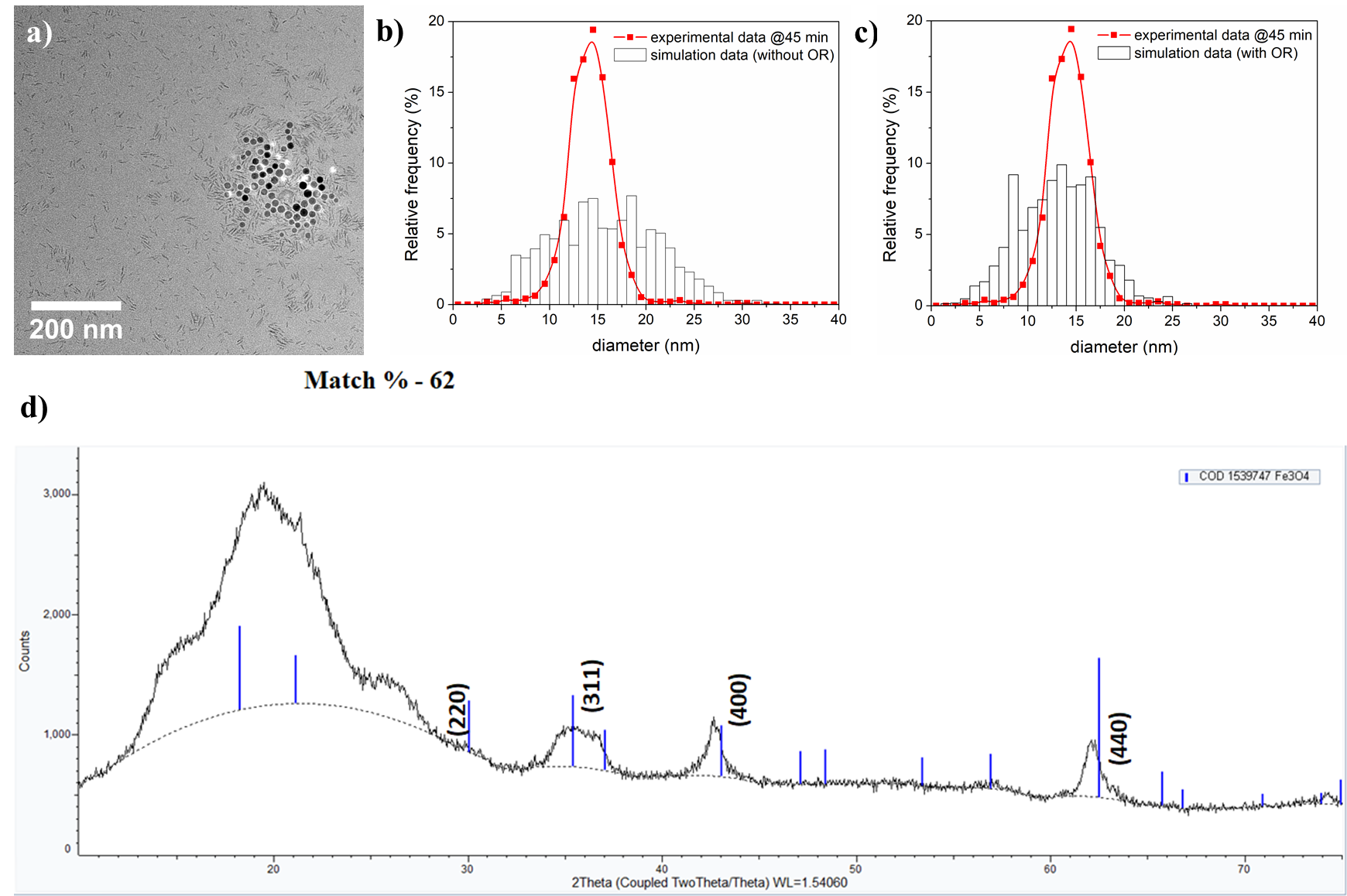


**Figure S1** a) HRTEM image of growth evolution of IONPs at 250 ^ο^C. Comparison between kMC simulation vs. experimental PSD of iron oxide formed through thermal decomposition route after 45 min of aging at 320 ˚C, b) without OR and c) with OR. d) XRD pattern of SNPs.

*4.2. Phase transfer and Functionalization of IONPs*


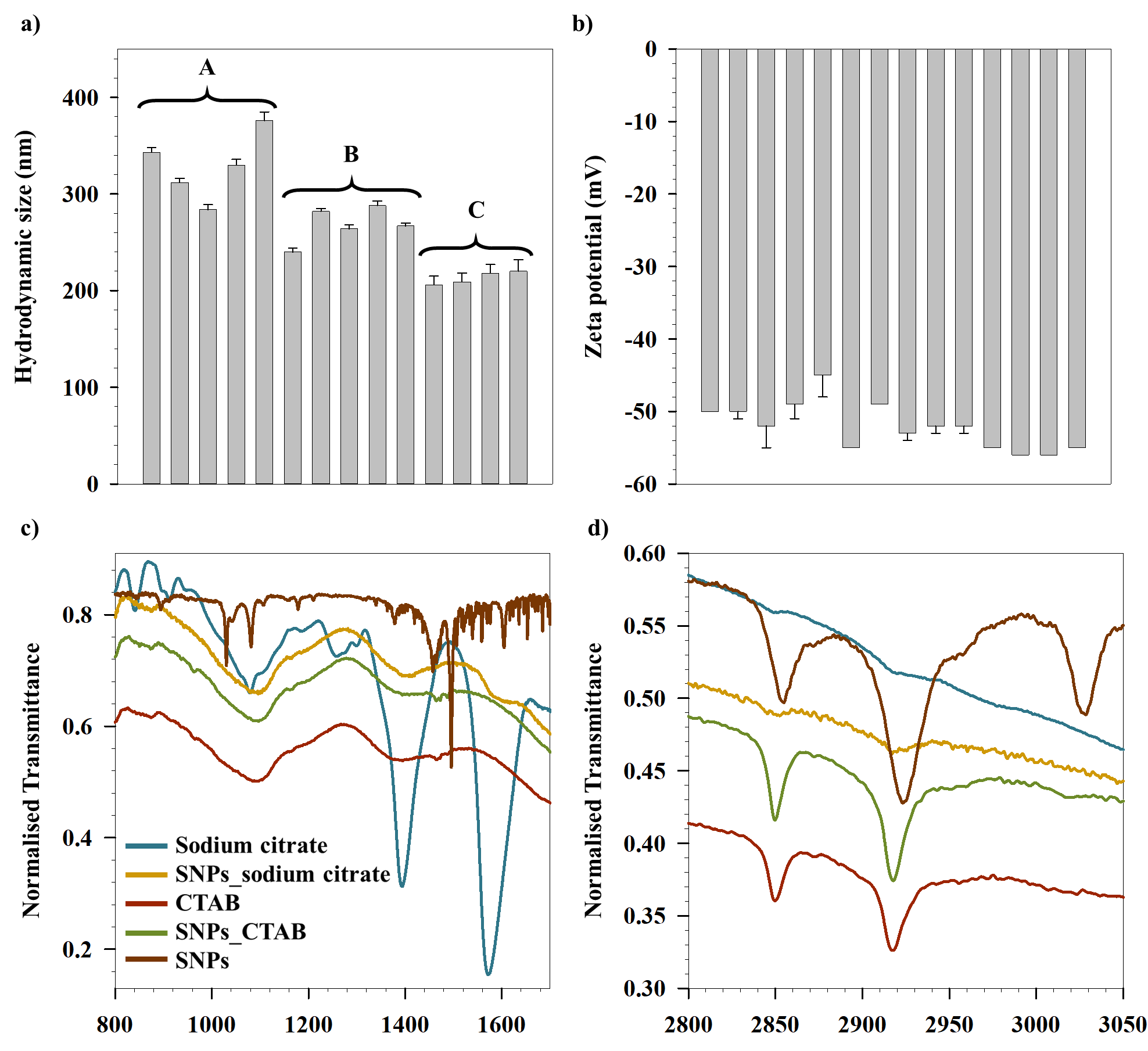


**Figure S2**. a) Size and b) zeta potential of SNPs phase transferred with Na-Citrate. ATR-FTIR spectra of sodium citrate, SNPs with sodium citrate, CTAB, SNPs with CTAB and SNPs in c) region 800-1600 cm-1 and d) region 2800-3200 cm-1.

*4.3. Influence of Magnetic Properties upon Functionalization*


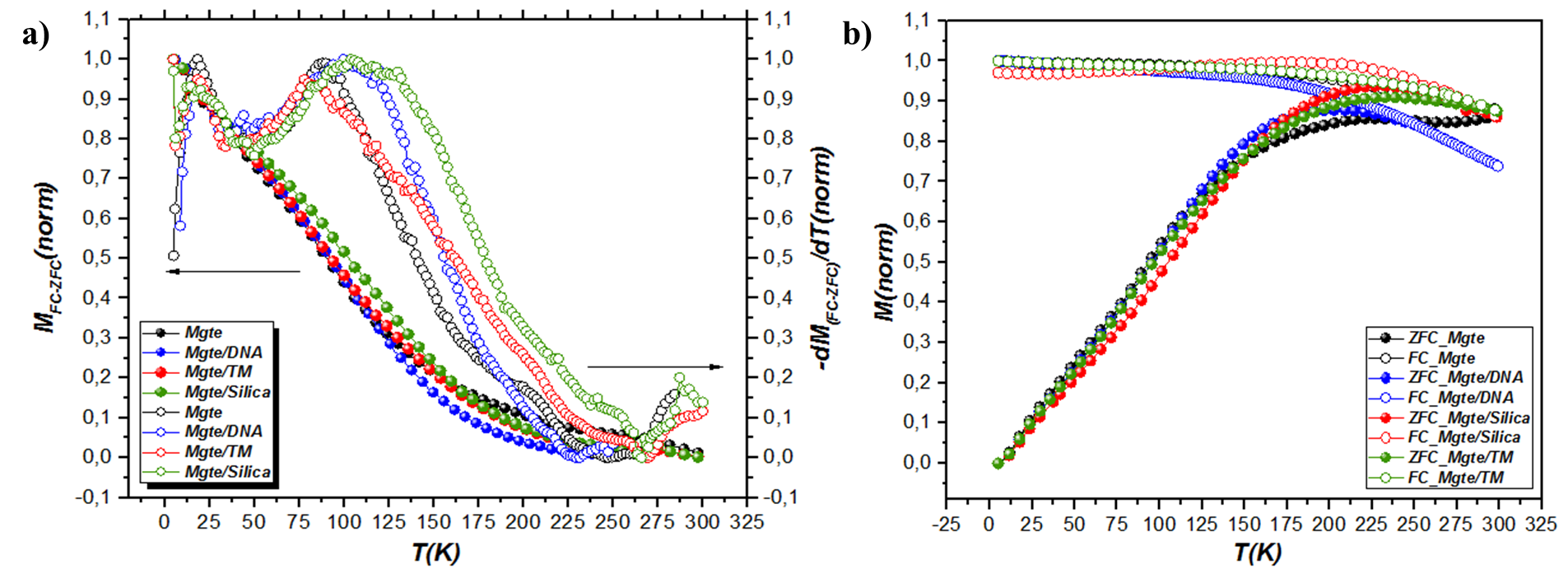


**Figure S3**. a) M_FC_ − M_ZFC_ and (dM_FC_ − M_ZFC_/dT) curves. b) ZFC/FC curves for Mag, Mag_DNA, Mag_TM and Mag_Silica.

In order to investigate magnetic nanoparticle interactions, low temperature remnant magnetization (5K) is carried out by the direct current demagnetization (DCD) and isothermal remnant magnetization (IRM) protocols (**Figure S4**).^[15, 16]^ In a typical DCD measurement, the sample is saturated in an external field of −5 T, then the external field is removed, and the remanence magnetization is recorded. The sample is then exposed to a reverse positive field (H_rev_) to invert the magnetization; so H_rev_ is removed and the remanence magnetization is recorded. The process is repeated several times, each time with a higher H_rev_, up to 5 T. IRM protocol is similar to DCD protocol but starting with the sample in a demagnetized state.^[17]^

M_r_^IRM^(H) and M_r_^DCD^(H) represent the same energy barriers in the case of an ensemble of non-interacting monodomain particles with uniaxial anisotropy. This is clearly expressed by the so-called Wohlfarth relationship:

$M_{r}^{DCD}(H)=1-2M_{r}^{IRM}(H)$ (S8)

Kelly et al. rewrote the Wohlfarth’s relationship in order to show more clearly the deviation of a real system from the non-interacting case:

$\delta M(H)=M_{r}^{DCD}-[1-2M_{r}^{IRM}]$ (S9)

where the δM parameter has zero value for a non-interacting system.^[18, 19]^ Interactions promoting the magnetized state such as exchange and other positive interactions lead to a positive value of δM parameter whereas negative deviation are due to demagnetizing interactions (e.g. dipole–dipole interactions).^[17]^


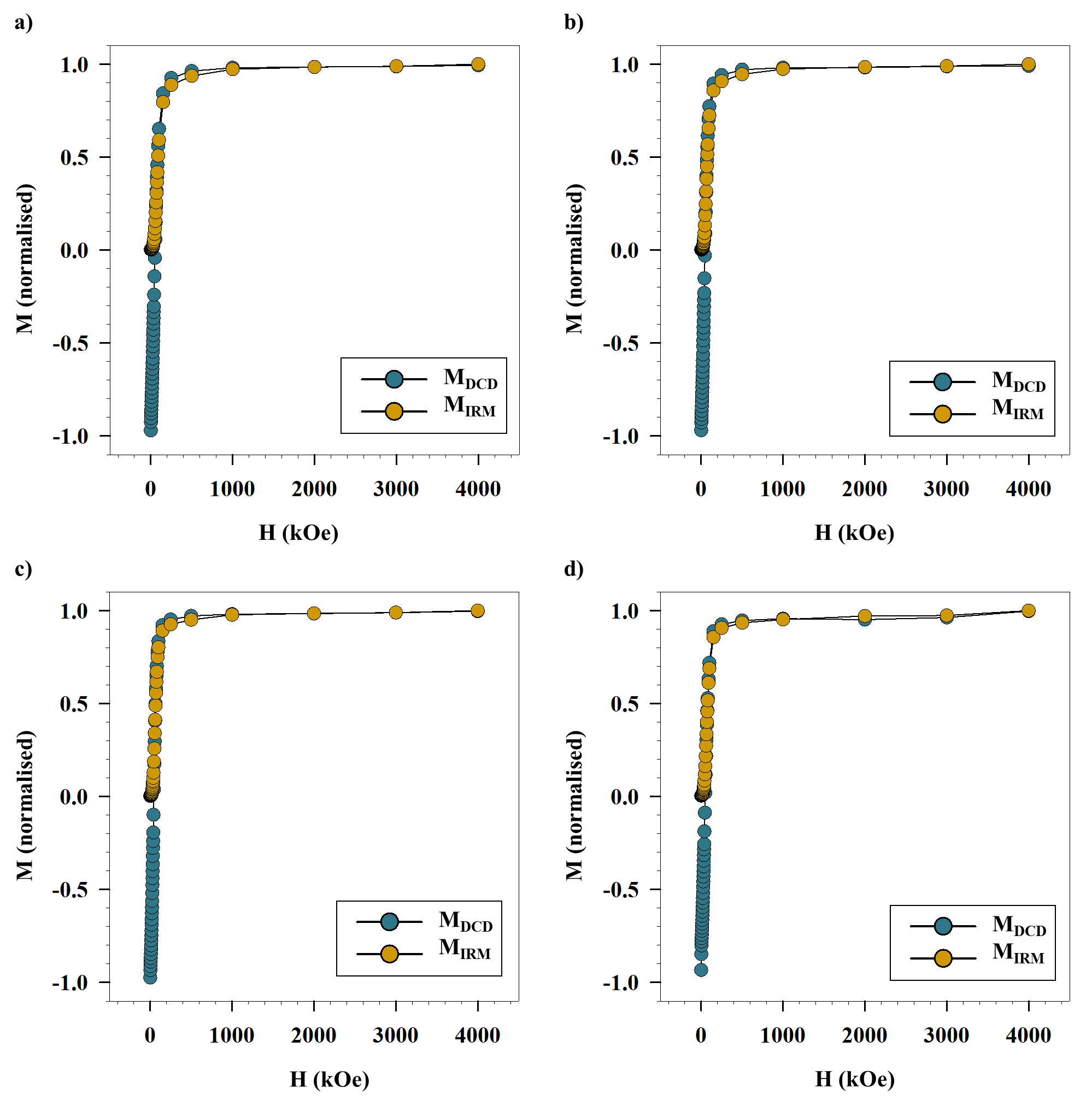


**Figure S4**. DCD and IRM curves for a) Mag, b) Mag_TMAPS, c) Mag_DNA amd d) Mag_Silica

**Figure S5**. Field dependence of magnetization recorded at 5K for CNPs.

**References**

1. Randolph, A., *Theory of particulate processes: analysis and techniques of continuous crystallization*. 2012: Elsevier.

2. Bandyopadhyaya, R., R. Kumar, and K. Gandhi, *Simulation of precipitation reactions in reverse micelles.* Langmuir, 2000. **16**(18): p. 7139-7149.

3. Ethayaraja, M., C. Ravikumar, D. Muthukumaran, K. Dutta, and R. Bandyopadhyaya, *CdS− ZnS Core− Shell Nanoparticle Formation: Experiment, Mechanism, and Simulation.* The Journal of Physical Chemistry C, 2007. **111**(8): p. 3246-3252.

4. Layek, A., G. Mishra, A. Sharma, M. Spasova, S. Dhar, A. Chowdhury, and R. Bandyopadhyaya, *A generalized three-stage mechanism of ZnO nanoparticle formation in homogeneous liquid medium.* The Journal of Physical Chemistry C, 2012. **116**(46): p. 24757-24769.

5. Bachhar, N. and R. Bandyopadhyaya, *Role of coating agent in iron oxide nanoparticle formation in an aqueous dispersion: Experiments and simulation.* Journal of colloid and interface science, 2016. **464**: p. 254-263.

6. Bachhar, N. and R. Bandyopadhyaya, *Predicting complete size distribution of nanoparticles based on interparticle potential: experiments and simulation.* The Journal of Physical Chemistry C, 2016. **120**(8): p. 4612-4622.

7. Treybal, R.E., *Mass transfer operations.* New York, 1980. **466**.

8. Friedlander, S.K., *Smoke, dust, and haze*. Vol. 198. 2000: Oxford University Press New York.

9. Kestin, J., M. Sokolov, and W.A. Wakeham, *Viscosity of liquid water in the range− 8 C to 150 C.* Journal of Physical and Chemical Reference Data, 1978. **7**(3): p. 941-948.

10. Oskam, G., Z. Hu, R.L. Penn, N. Pesika, and P.C. Searson, *Coarsening of metal oxide nanoparticles.* Physical Review E, 2002. **66**(1): p. 011403.

11. Kabalnov, A.S. and E. Schchukin, *Ostwald ripening theory: applications to fluorocarbon emulsion stability.* Advances in colloid and interface science, 1992. **38**: p. 69-97.

12. Kim, J., H.S. Kim, N. Lee, T. Kim, H. Kim, T. Yu, I.C. Song, W.K. Moon, and T. Hyeon, *Multifunctional uniform nanoparticles composed of a magnetite nanocrystal core and a mesoporous silica shell for magnetic resonance and fluorescence imaging and for drug delivery.* Angewandte Chemie International Edition, 2008. **47**(44): p. 8438-8441.

13. Bandyopadhyay, S., M.K. Andersen, M.A.A. Alvi, A. Sharma, R. Raju, B.H. McDonagh, and W.R. Glomm, *Incorporation of Fe@ Au nanoparticles into multiresponsive pNIPAM-AAc colloidal gels modulates drug uptake and release.* Colloid and Polymer Science, 2016. **294**(12): p. 1929-1942.

14. Peeters, K., G. Lespes, T. Zuliani, J. Ščančar, and R. Milačič, *The fate of iron nanoparticles in environmental waters treated with nanoscale zero-valent iron, FeONPs and Fe3O4NPs.* Water research, 2016. **94**: p. 315-327.

15. Laureti, S., G. Varvaro, A. Testa, D. Fiorani, E. Agostinelli, G. Piccaluga, A. Musinu, A. Ardu, and D. Peddis, *Magnetic interactions in silica coated nanoporous assemblies of CoFe2O4 nanoparticles with cubic magnetic anisotropy.* Nanotechnology, 2010. **21**(31): p. 315701.

16. Muscas, G., N. Yaacoub, G. Concas, F. Sayed, R.S. Hassan, J.-M. Greneche, C. Cannas, A. Musinu, V. Foglietti, and S. Casciardi, *Evolution of the magnetic structure with chemical composition in spinel iron oxide nanoparticles.* Nanoscale, 2015. **7**(32): p. 13576-13585.

17. Peddis, D., P.E. Jönsson, S. Laureti, and G. Varvaro, *Magnetic interactions: A tool to modify the magnetic properties of materials based on nanoparticles*, in *Frontiers of Nanoscience*. 2014, Elsevier. p. 129-188.

18. Wohlfarth, E.P., *Relations between different modes of acquisition of the remanent magnetization of ferromagnetic particles.* Journal of Applied Physics, 1958. **29**(3): p. 595-596.

19. Kelly, P., K. O'grady, P. Mayo, and R. Chantrell, *Switching mechanisms in cobalt-phosphorus thin films.* IEEE Transactions on Magnetics, 1989. **25**(5): p. 3881-3883.
